# Disrupting FKF1 homodimerization increases *FT* transcript levels in the evening by enhancing CO stabilization

**DOI:** 10.1007/s00299-024-03207-w

**Published:** 2024-04-18

**Authors:** Sung Won Cho, Jameela Lokhandwala, Jun Sang Park, Hye Won Kang, Mingi Choi, Hong-Quan Yang, Takato Imaizumi, Brian D. Zoltowski, Young Hun Song

**Affiliations:** 1 Department of Biology, Ajou University, Suwon, Korea; 2 Institute of Agricultural Life Sciences, Seoul National University, Seoul, Korea; 3 Department of Chemistry, Southern Methodist University, Dallas, TX USA; 4 Department of Agricultural Biotechnology, Seoul National University, Seoul, Korea; 5 Shanghai Key Laboratory of Plant Molecular Sciences, College of Life Sciences, Shanghai Normal University, Shanghai, China; 6 Department of Biology, University of Washington, Seattle, WA USA; 7 Plant Genomics and Breeding Institute, Seoul National University, Seoul, Korea

**Keywords:** Flowering time, FLAVIN-BINDING, KELCH REPEAT, F-BOX 1, CONSTANS, CONSTITUTIVE PHOTOMORPHOGENIC 1, FLOWERING LOCUS T, Dimerization

## Abstract

**Key message:**

FKF1 dimerization is crucial for proper *FT* levels to fine-tune flowering time. Attenuating FKF1 homodimerization increased CO abundance by enhancing its COP1 binding, thereby accelerating flowering under long days.

**Abstract:**

In Arabidopsis (*Arabidopsis thaliana*), the blue-light photoreceptor FKF1 (FLAVIN-BINDING, KELCH REPEAT, F-BOX 1) plays a key role in inducing the expression of *FLOWERING LOCUS T* (*FT*), encoding the main florigenic signal in plants, in the late afternoon under long-day conditions (LDs) by forming dimers with *FT* regulators. Although structural studies have unveiled a variant of FKF1 (FKF1 I160R) that disrupts homodimer formation in vitro, the mechanism by which disrupted FKF1 homodimer formation regulates flowering time remains elusive. In this study, we determined that the attenuation of FKF1 homodimer formation enhances *FT* expression in the evening by promoting the increased stability of CONSTANS (CO), a primary activator of *FT*, in the afternoon, thereby contributing to early flowering. In contrast to wild-type FKF1, introducing the FKF1 I160R variant into the *fkf1* mutant led to increased *FT* expression under LDs. In addition, the FKF1 I160R variant exhibited diminished dimerization with FKF1, while its interaction with GIGANTEA (GI), a modulator of FKF1 function, was enhanced under LDs. Furthermore, the FKF1 I160R variant increased the level of CO in the afternoon under LDs by enhancing its binding to COP1, an E3 ubiquitin ligase responsible for CO degradation. These findings suggest that the regulation of FKF1 homodimerization and heterodimerization allows plants to finely adjust *FT* expression levels around dusk by modulating its interactions with GI and COP1.

**Supplementary Information:**

The online version contains supplementary material available at 10.1007/s00299-024-03207-w.

## Introduction

The timely flowering of plants in the appropriate seasons is crucial for their reproductive fitness (Song et al. 2015; Exposito-Alonso 2020). In many plant species, seasonal flowering is regulated by the expression of *FLOWERING LOCUS T* (*FT*), which encodes a mobile florigenic signal (Corbesier et al. 2007; Jaeger and Wigge 2007; Mathieu et al. 2007). FT protein is produced in the vasculature of leaves and travels to the shoot apical meristem (Corbesier et al. 2007; Jaeger and Wigge 2007; Mathieu et al. 2007; Takagi et al. 2023). In Arabidopsis (*Arabidopsis thaliana*), a facultative long-day plant, *FT* is strongly expressed under long-day conditions (LDs), thereby promoting flowering (Suárez-López et al. 2001). Under LDs, *FT* expression largely relies on the function of the transcriptional activator CONSTANS (CO), whose stabilization during the daytime is controlled by photoreceptors: This regulation determines the timing of flowering (Samach et al. 2000; Valverde et al. 2004; Song et al. 2015; Takagi et al. 2023).

Under conventional laboratory LD conditions, *FT* mRNA abundance only peaks at the end of the day (Suárez-López et al. 2001). However, under natural LDs, in which a simulated red-to-far-red ratio mimicking that of sunlight and daily temperature cycles is superimposed onto LDs, the daily expression of *FT* peaks in both the morning and evening (Song et al. 2018; Kim et al. 2024). This bimodal expression pattern is determined by the regulation of CO stability and CO-independent regulatory pathways (Song et al. 2018; Kim et al. 2024; Lee et al. 2023; Takagi et al. 2023). Unlike *FT* expression, the daily profile of CO levels shows two peaks under both conventional laboratory LDs and natural LDs: a narrow peak early in the morning and a broad peak in the late afternoon (Valverde et al. 2004; Song et al. 2012, 2018). During the morning under natural LDs, CO is stabilized by the activity of the far-red-light-responsible photoreceptor phytochrome A (phyA) (Song et al. 2018). EARLY FLOWERING 3 (ELF3), an interaction partner of CONSTITUTIVE PHOTOMORPHOGENIC 1 (COP1), forms a protein complex with CO and mediates its degradation (Jang et al. 2008; Song et al. 2018). PhyA interacts with ELF3 in the morning (Song et al. 2018), which might interfere with the function of ELF3 in destabilizing CO. By contrast, the red-light-activated phyB, the major red-light photoreceptor in plants, facilitates the destabilization of CO by forming a complex with CO and HOS1 (HIGH EXPRESSION OF OSMOTICALLY RESPONSIVE GENES 1), an E3 ubiquitin ligase that degrades its proteolytic targets such as CO (Lazaro et al. 2015). In addition, the F-box blue-light photoreceptor ZEITLUPE (ZTL), which possesses E3 ubiquitin ligase activity, binds to and degrades CO during the morning (Song et al. 2014).

In the afternoon under conventional laboratory LDs, FKF1 (FLAVIN-BINDING, KELCH REPEAT, F-BOX 1), a ZTL homologous protein, strongly promotes the stability of CO by interacting with CO through its LOV (Light, Oxygen, or Voltage) domain (Song et al. 2012). Since the LOV domain is responsible for blue-light absorption, the FKF1–CO interaction increases under blue light (Ito et al. 2012; Song et al. 2012). Moreover, FKF1 binds to COP1 and inhibits its homodimer formation, leading to a decrease in COP1-dependent CO degradation (Lee et al. 2017). In addition to FKF1, CRYPTOCHROME 2 (CRY2), another type of blue-light photoreceptor, also contributes to CO stabilization in the afternoon. CRY2 interacts with COP1 and SUPRESSOR OF PHYA-105 1 (SPA1) under blue light and suppresses the formation of a complex between COP1 and SPA1, resulting in the attenuation of CO degradation mediated by the E3 ubiquitin ligase activity of COP1 (Jang et al. 2008; Zuo et al. 2011).

Among photoreceptors, two homologous blue-light receptors, ZTL and FKF1, exhibit contrasting functions not only in regulating CO stability but also in regulating the seasonal expression of *FT* (Takagi et al. 2023). Under natural LDs, ZTL forms a protein complex with the *FT* inhibitors TARGET OF EAT1 (TOE1) and TOE2, which specifically inhibits *FT* expression in the morning (Zhang et al. 2015; Kim et al. 2024). In contrast to ZTL, FKF1 promotes *FT* transcription more prominently in the afternoon under natural LDs (Song et al. 2018). Besides stabilizing CO protein, FKF1 induces *FT* expression by removing CYCLING DOF FACTOR 1 (CDF1), a transcriptional repressor of *CO* and *FT*, from the *FT* promoter in the afternoon via its E3 ubiquitin ligase activity (Imaizumi et al. 2005; Sawa et al. 2007; Song et al. 2012). The diel accumulation patterns of FKF1 and ZTL are remarkably similar, with both peaking in the afternoon (Kim et al. 2024). However, ZTL is more abundant in the morning than FKF1 (Kim et al. 2024). The regulation of *FT* expression by the opposing functions of FKF1 and ZTL involves dimer formation between these proteins and the role of their partner protein GIGANTEA (GI). Higher abundance of ZTL in the morning inhibits the activity of FKF1 in promoting flowering through heterodimer formation (Takase et al. 2011; Hwang et al. 2019). Under LDs, FKF1 and ZTL interact with GI in a blue-light-dependent manner in the afternoon through their LOV domains. These interactions play crucial roles in inactivating ZTL during this time window and facilitating FKF1-induced CDF1 degradation (Kim et al. 2007; Sawa et al. 2007; Hwang et al. 2019). Consequently, the binding of GI to ZTL leads to the sequestration of FKF1, as well as CO, from ZTL, resulting in the stabilization of CO and the induction of *FT* expression by FKF1 in the afternoon (Hwang et al. 2019).

Structural studies have demonstrated that the LOV domains of FKF1 and ZTL intrinsically form homodimers (Nakasako et al. 2005; Pudasaini et al. 2017; Kwon et al. 2022). However, upon binding to GI, the LOV homodimers are converted to monomers (Kwon et al. 2022). Considering their roles in modulating CO stability, these observations suggest that the monomeric and dimeric states of FKF1 and ZTL (determined via their LOV domains) might be crucial for their functions in regulating flowering time (Song et al. 2012, 2014; Hwang et al. 2019). Although we previously demonstrated that the Ile160 residue in the FKF1 LOV domain is important for homodimer formation in vitro (Pudasaini et al. 2017), the biological relevance of conformational changes in FKF1 in regulating flowering time remains unclear.

In this study, to explore the effects of structural changes in FKF1 on flowering time in Arabidopsis at the molecular level, we investigated the properties associated with the regulation of CO stabilization and *FT* gene expression using the FKF1 I160R variant, which shows reduced homodimer formation. Our findings reveal the altered binding affinities between FKF1 and complex-forming proteins, confirming their potential to finely regulate flowering time in plants at the mechanistic level.

## Materials and methods

### Plant materials and growth conditions

All *Arabidopsis thaliana* plants [WT, *fkf1* (Nelson et al. 2000), *FKF1:HA-FKF1/fkf1* (Imaizumi et al. 2003), *FKF1:HA-FKF1 GI:GI-TAP/fkf1 gi-2* (Sawa et al. 2007), *GI:GI-TAP/gi-2* (David et al. 2006)*, CO:HA-CO* (Song et al. 2012)*, FKF1:HA-FKF1 I160R/fkf1, FKF1:FKF1 I160R GI:GI-TAP/fkf1 gi-2, CO:HA-CO/cop1-4, FKF1:HA-FKF1 I160R CO:HA-CO/fkf1*] used in this study are the Columbia (Col-0) ecotype.

The I160R mutation in the *FKF1* gene was introduced using the FKF1 I160R reverse primer (5ʹ-GAAGACCTGTATCCCACGTACGTGTGTAATGGT-3ʹ, the underline indicates the sequences that encode arginine).

Plants were grown on soil in a plant growth room for flowering time measurement and tobacco transient expression. For Arabidopsis co-immunoprecipitation assays as well as protein and gene expression analyses, seeds were sterilized, stratified at 4 °C for 3 days, and sown onto 0.5X Murashige Skoog (MS) media (Duchefa Biochemie) with 1% (w/v) sucrose.

Seedlings were grown in LDs (16-h light/8-h dark) with constant 22 °C under full-spectrum white LED light (4,470 K) (Bissol LED) with a fluence rate of 80–100 μmol m^−2^ s^−1^. For light quality treatment, plants grown in LDs were transferred to LDs with monochromatic blue (450 nm) or red (660 nm) ranging from 40 to 50 μmol m^−2^ s^−1^ on day 8 and harvested at ZT12 on day 10.

To analyze the flowering time of *Arabidopsis*, the number of rosette and cauline leaves on the main stem were counted when the inflorescence reached 3 cm long after bolting. The analysis of flowering time was carried out independently in triplicate, involving 12 individual plants per trial. Each iteration of the experiment yielded consistent and comparable results.

### RNA isolation and gene expression analysis

For diel gene expression analysis, seedlings grown on agar plates under LDs for 10 days were harvested at 3 h intervals from ZT1 to ZT22. Total RNA was extracted using the Higene Total RNA Prep Kit (BioFact). Subsequently, 2 μg of isolated RNA was employed to synthesize first-strand cDNA using the DiastarTM RT kit (SolGent) with an oligo dT primer. Following dilution with 40 μl of water, 2 μl of cDNA was utilized for quantitative polymerase chain reaction (qPCR) performed on a CFX96 thermal real-time cycler (Bio-Rad). Primers and PCR conditions for *FKF1*, *CO*, *FT*, and *IPP2* were previously detailed (Song et al. 2012). Relative expression levels were determined by normalizing against *IPP2* expression.

### Immunoblot analysis and protein quantification

To analyze FKF1, FKF1 I160R, and CO protein abundance, *FKF1:HA-FKF1/fkf1, FKF1:HA-FKF1 I160R/fkf1, CO:HA-CO*, and *FKF1:HA-FKF1 I160R CO:HA-CO/fkf1* transgenic plants were grown on agar plates under LDs. Seedlings were collected at 4-h intervals from ZT0 to ZT20 on day 10, with an additional harvest at ZT0.5 for CO. Protein extraction, nuclei isolation, and Western blot procedures were previously described (Song et al. 2012). In brief, total proteins were extracted using an extraction buffer [50 mM sodium-phosphate pH 7.4, 100 mM NaCl, 10% glycerol, 5 mM EDTA, 1% NP-40, 0.5% SDS, 0.5% sodium deoxycholate, 50 μM MG-132, and protease inhibitor tablets-EDTA free (Roche)]. Nuclei proteins were obtained using the CelLytic Plant Nuclei Isolation/Extraction Kit (Sigma) following the manufacturer's protocol. Detection of HA-FKF1 and HA-CO proteins was accomplished using the anti-HA antibody (3F10, Roche). Actin and Histone H3 served as internal loading controls for whole protein extract and nuclei proteins, respectively, and were detected using anti-actin (AC009, ABclonal) and anti-histone H3 (ab1791, Abcam) antibodies, respectively. For protein quantification, immunoreactive proteins were visualized with SuperSignal West Pico (Thermo Fisher) and/or ECL select Femto (Bio-Rad) mixture solution, followed by imaging using the ChemiDocTM Touch imaging system (Bio-Rad). The captured image was analyzed for quantification using the Image Lab software (Bio-Rad). Relative amounts of HA-fusion proteins were normalized based on the expression values of actin and Histone H3 for HA-FKF1 and HA-CO, respectively.

### Co-immunoprecipitation experiments

The co-immunoprecipitation assays were conducted following previously established procedures (Song et al. 2012). For the examination of FKF1 homodimerization and interactions involving FKF1 with ZTL, as well as FKF1 with CO in tobacco cells, agrobacteria strain GV3101 carrying *35S:FKF1-TAP*, *35S:HA-FKF1*, *35S:HA-FKF1 I160R*, *35S:ZTL-TAP*, or *35S:CO-TAP* constructs were infiltrated into 3- to 4-week-old *Nicotiana benthamiana* leaves. Subsequently, the tobacco plants were grown under LDs for 3 days, and the infiltrated leaves were harvested to obtain whole protein extracts. An equivalent amount of ground tissues was utilized for the co-immunoprecipitation experiment. To investigate in vivo interactions, *GI:GI-TAP*, *FKF1:HA-FKF1/fkf1*, *FKF1:HA-FKF1 I160R/fkf1 #2–3*, *FKF1:HA-FKF1 GI:GI-TAP/fkf1 gi-2*, *FKF1:HA-FKF1 I160R GI:GI-TAP/fkf1 gi-2*, and *CO:HA-CO/cop1-4* plants were grown under the specified conditions. All co-immunoprecipitation experiments were conducted using 10-day-old seedlings and were repeated three times with independently harvested samples. The proteins GI-TAP, HA-FKF1, and COP1 were detected through Western blotting using anti-protein A (Sigma), anti-HA (3F10, Roche), and anti-COP1 (Lian et al. 2011) antibodies, respectively.

### Expression of the FKF1 I160R protein

An FKF1 I160R variant composed of residues 28–174 was previously cloned into a pGST-Parallel vector using the NcoI and XhoI cut sites (Pudasaini et al. 2017). 24 L of FKF1 I160R proteins were expressed in *E. coli* JM109DE3. JM109DE3 cells transformed with pGST-FKF1 I160R were grown at 37 °C until an OD_600_ of 0.4–0.5, and the temperature was reduced to 18 °C for 40 min prior to induction with 0.3 mM IPTG (RPI). Cells were harvested after about 20 h and pelleted in a buffer containing 50 mM Hepes (pH 8.0), 100 mM NaCl, and 10% glycerol and stored at – 80 °C.

### Purification of the FKF1 I160R protein

The pellet from a 24-l expression of FKF1 I160R proteins was thawed and lysed via sonication. The lysate was clarified via centrifugation at 22,000 RPM for 1 h. The supernatant was applied to glutathione affinity resin (Qiagen) and purified in the light to enhance protein stability. After binding, GST-tagged FKF1 I160R was treated on a column with 4 mg of TEV protease at 22 °C for 2 h. Proteins were then eluted by the addition of buffer containing 50 mM Hepes (pH 8.0), 100 mM NaCl, and 10% glycerol. Eluted protein was concentrated to 5 ml and further purified by Size Exclusion Chromatography using a Superdex S200 column equilibrated in the same buffer. Fractions containing FKF1 I160R proteins were pooled together, and concentrated to 500 μl, resulting in a final overall yield of about 0.3 mg of FKF1 I160R proteins.

### Kinetic characterization of the FKF1 I160R variant

For kinetic characterization, the purified FKF1 I160R protein was diluted in a buffer containing 50 mM Hepes (pH 8.0), 100 mM NaCl, 10% glycerol, and 1.0 M imidazole (pH 8.0) to a final imidazole concentration of 300 mM to accelerate the recovery kinetics. A 100 μl sample was placed into a 1.0 cm pathlength quartz cuvette, and spectra were recorded every 1800 s for 24 h. Kinetics were extracted from the absorbance trace at 450 nm through a single exponential fit.

### Statistical analysis

Statistical analyses, including calculations of mean, standard error of the mean (SEM), and *t* tests, were conducted using Microsoft Excel software. The significance of differences between two experimental groups was determined using a two-tailed Student’s *t* test. Significance levels were set at *P* values of < 0.05, < 0.01, and < 0.001 for assessing the significance of differences between data sets.

## Results

### The FKF1 I160R variant shows enhanced activity in regulating flowering time

The blue-light photoreceptor FKF1 is pivotal in the regulation of photoperiod-dependent flowering (Song et al. 2015). The LOV domain within FKF1 is indispensable for its functionality, where the light-dependent reversible formation of a Flavin-C4a within the LOV domain regulates function, and the LOV domain mediates dimer formation. Mutations within the LOV domain compromise its interactions with GI and CO *in planta* under blue-light conditions (Sawa et al. 2007; Song et al. 2012; Pudasaini et al. 2017). Moreover, the LOV domain forms a homodimer through equivalent anti-parallel contacts following the β-scaffold (Pudasaini et al. 2017). The I160R variant of the FKF1 LOV domain, characterized by a mutation at position Ile160 within one of the β-sheets, disrupts FKF1 LOV homodimer formation in vitro (Pudasaini et al. 2017), suggesting that this amino acid residue may be crucial for determining the homodimeric and heterodimeric states of FKF1 in Arabidopsis. Notably, the I160R variant demonstrates photochemistry consistent with WT FKF1 (Supplementary Fig. S1), thereby allowing one to isolate the biological effects of homo/hetero dimerization independent of photochemical function. Therefore, we explored the effect of changes in dimer formation on the role of FKF1 in regulating flowering time, focusing on the I160R mutation. Considering that FKF1 predominantly accumulates in the afternoon and that its function is specifically linked to the evening peak levels of *FT* rather than the morning *FT* peak (Imaizumi et al. 2003; Song et al. 2018), we opted to employ conventional laboratory LDs, hereafter referred to as LDs, instead of natural LDs. We generated the *FKF1:HA-FKF1 I160R* expression cassette, designed to express the full-length FKF1 I160R with an N-terminal hemagglutinin (HA) tag, driven by the *FKF1* promoter, and introduced this construct into the Arabidopsis *fkf1* mutant (Imaizumi et al. 2003).

**Fig. 1:**
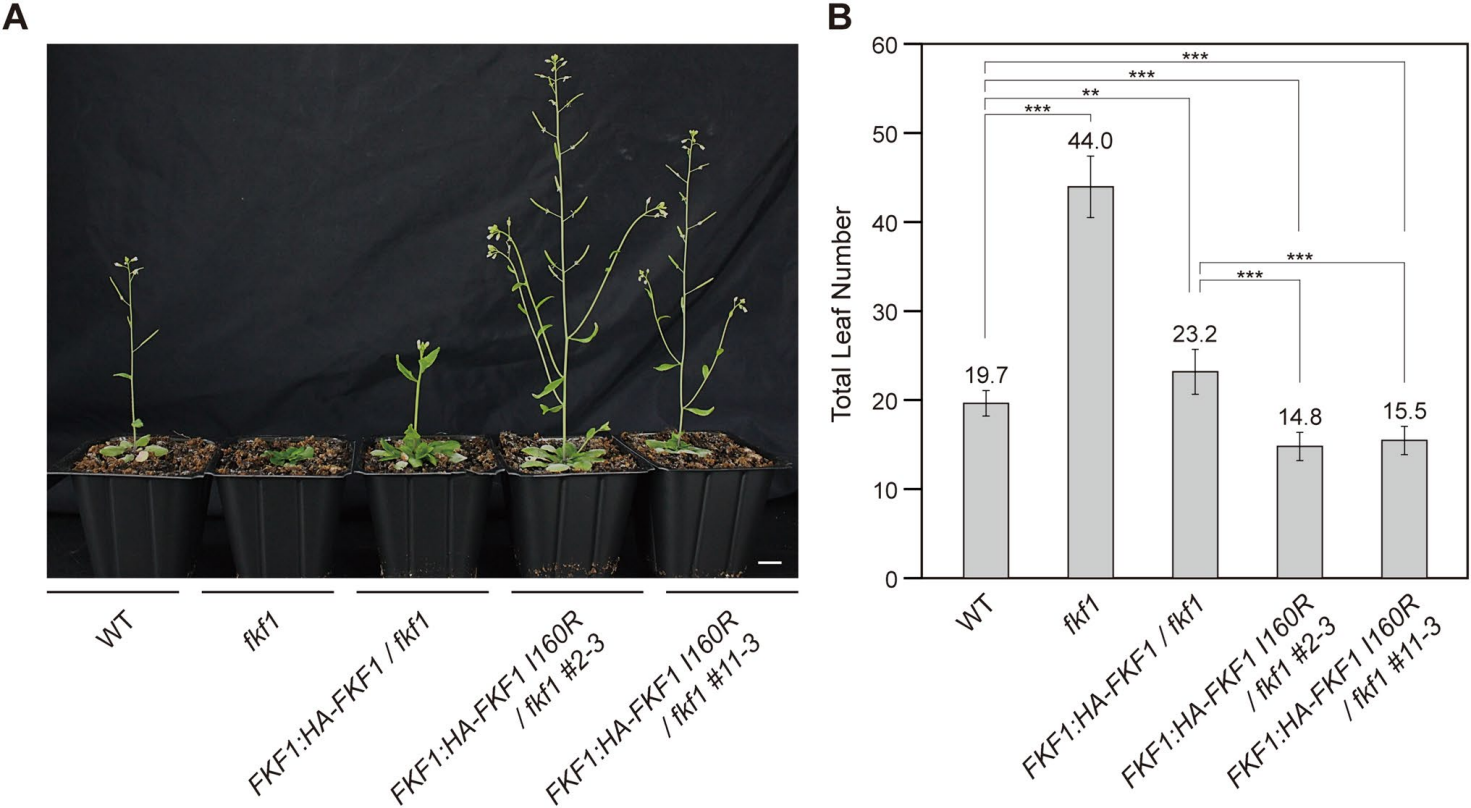
Complementation of the *fkf1* mutant with the FKF1 I160R variant promotes flowering. **A** Flowering phenotypes of representative 5- to 6-week-old *Arabidopsis thaliana* plants (Col-0 ecotype) grown under long-day conditions (LDs). Scale bar is 1 cm. **B** Total leaf number at bolting of wild-type (WT, Col-0 ecotype), *fkf1* mutant, *FKF1:HA-FKF1/fkf1*, and *FKF1:HA-FKF1 I160R/fkf1 #2-3*, and *FKF1:HA-FKF1 I160R/fkf1 #11-3* plants. The data underwent statistical analysis using a two-tailed Student's *t* test. **p < 0.01 and ***p ≤ 0.001. Data represent means ± SEM

We compared the effects of the wild-type version of FKF1 and the FKF1 I160R variant on complementing the late-flowering phenotype of the *fkf1* mutant by measuring total leaf number at bolting. Under LDs, wild-type and *fkf1* plants bolted with an average leaf number of 19.7 and 44.0, respectively (Fig. 1A, B). Wild-type FKF1 nearly complemented the late flowering of the *fkf1* mutant, with 23.2 leaves at bolting (Fig. 1A, B). Notably, two transgenic lines of *FKF1:HA-FKF1 I160R/fkf1* flowered significantly earlier than wild-type plants (Fig. 1A, B). These results indicate that the I160R mutation in FKF1 enhances its function in regulating flowering time.

### The FKF1 I160R variant exhibits reduced protein stability but enhanced efficacy in inducing *FT* expression

**Fig. 2:**
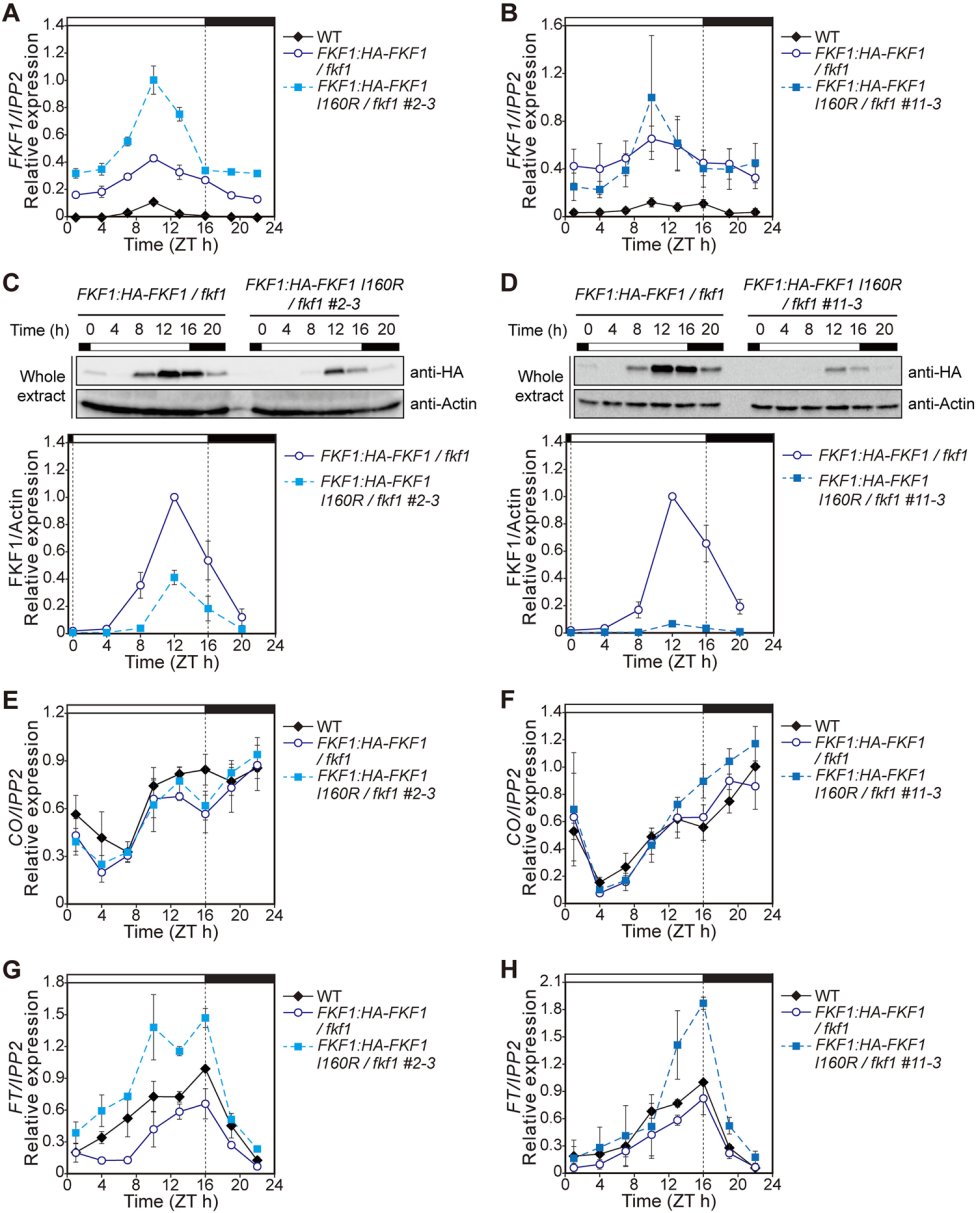
Introducing the FKF1 I160R variant into the *fkf1* mutant increases *FT* mRNA levels. Comparative analysis of the levels of *FKF1* mRNA (**A**, **B**), FKF1 protein (**C**, **D**), *CO* mRNA (**E**, **F**), and *FT* mRNA (**G**, **H**) in WT, *FKF1:HA-FKF1/fkf1*, and *FKF1:HA-FKF1I160R/fkf1 #2-3* (**A**, **C**, **E**, **G**), and *FKF1:HA-FKF1 I160R/fkf1 #11-3* (**B**, **D**, **F**, **H**) plants grown under LDs. For gene expression analysis, plants were harvested every 3 h from ZT1 to ZT22 on day 10. *FKF1*, *CO*, and *FT* transcript levels were normalized to *IPP2* levels. To compare protein abundance, plants were collected every 4 h from ZT0 to ZT20 on day 10. HA-FKF1 proteins were detected by immunoblotting with anti-HA antibody. Relative protein abundance was normalized to Actin levels. Data represent means ± SEM from at least three independent biological replicates

We investigated the molecular characteristics of the FKF1 I160R variant associated with the promotion of flowering. *FKF1* mRNA levels and FKF1 protein abundance exhibit daily rhythms, with peak levels in the afternoon under LDs (Imaizumi et al. 2003). In two *FKF1:HA-FKF1 I160R/fkf1* transgenic lines, the diel profile of *FKF1 I160R* mRNA under LDs was either similar to or higher than that of *FKF1* mRNA in *FKF1:HA-FKF1/fkf1* plants, with peak levels at Zeitgeber Time 10 (ZT10), resembling that of wild-type plants (Fig. 2A, B). We previously observed that introducing the I160R mutation into a truncated version of FKF1 comprising amino acid residues 28–174 of the LOV domain led to a transition from homodimer to monomer formation and increased susceptibility to protein degradation in vitro (Pudasaini et al. 2017). Consistent with this observation in vitro, FKF1 I160R protein levels in *FKF1:HA-FKF1 I160R/fkf1* plants were much lower than FKF1 protein levels in *FKF1:HA-FKF1/fkf1* plants while exhibiting the same daily rhythm (Fig. 2C, D), confirming the finding that the FKF1 I160R variant is more susceptible to proteolysis than the wild-type form (Pudasaini et al. 2017). These results suggest that changes in the abundance of FKF1 protein may not be the primary factor contributing to the early flowering of *FKF1:HA-FKF1I160R/fkf1* plants.

Under LDs, FKF1 induces *FT* mRNA expression in the evening through multiple feedforward mechanisms that promote flowering (Song et al. 2012). In addition to its role in CO stabilization, FKF1 promotes the expression of *CO* and *FT* in the afternoon by directly alleviating their transcriptional repression imposed by CDF proteins via its E3 ubiquitin ligase activity (Imaizumi et al. 2005; Sawa et al. 2007; Fornara et al. 2009; Song et al. 2012). Therefore, we examined *CO* and *FT* transcript levels under LDs. In both *FKF1:HA-FKF1 I160R/fkf1* lines, *CO* mRNA levels were comparable to those in wild-type and *FKF1:HA-FKF1/fkf1* plants (Fig. 2E, F). In contrast to *CO*, there was a notable increase in the abundance of *FT* mRNA in *FKF1:HA-FKF1 I160R/fkf1* lines (Fig. 2G, H). This increase appeared to correlate with the protein levels of the FKF1 I160R variant (Fig. 2C, D). These findings suggest that the FKF1 I160R variant selectively regulates *FT* over *CO* expression. Considering the simultaneous inhibition of *CO* and *FT* expression by CDF1 (Song et al. 2012), these findings further support the notion that this regulatory mechanism is likely unrelated to the degradation of CDF by FKF1.

### The FKF1 I160R variant displays increased interaction with GI but decreased binding to ZTL

The transition between monomeric and dimeric states appears to be a critical factor for the role of FKF1 in regulating flowering time. FKF1 has an inherent capacity to form a homodimer but is converted into a monomer upon binding with GI (Kwon et al. 2022). Under LDs, FKF1 forms a complex with GI in the afternoon. This complex formation enhances the E3 ubiquitin ligase activity of FKF1 by facilitating the recognition of its proteolytic targets, CDF proteins, resulting in the induction of *CO* and *FT* expression during this time period (Sawa et al. 2007). Conversely, under short-day conditions, the interaction between FKF1 and GI is impaired during the daytime, contributing to very low expression levels of *CO* and *FT* (Sawa et al. 2007). The I160R mutation abolished the homodimerization of FKF1 via its LOV domain in vitro (Pudasaini et al. 2017). However, the I160 site would not disrupt the FKF1-GI interface predicted from the structure of GI bound to LKP2 (Kwon et al. 2022 and Supplementary Fig. S2). These observations suggest that the heightened functionality of FKF1 in elevating *FT* mRNA levels due to the I160R mutation could be attributed to changes in the dimerization state of FKF1. Consequently, we investigated the effect of the I160R mutation on FKF1 dimerization in plant cells.

**Fig. 3:**
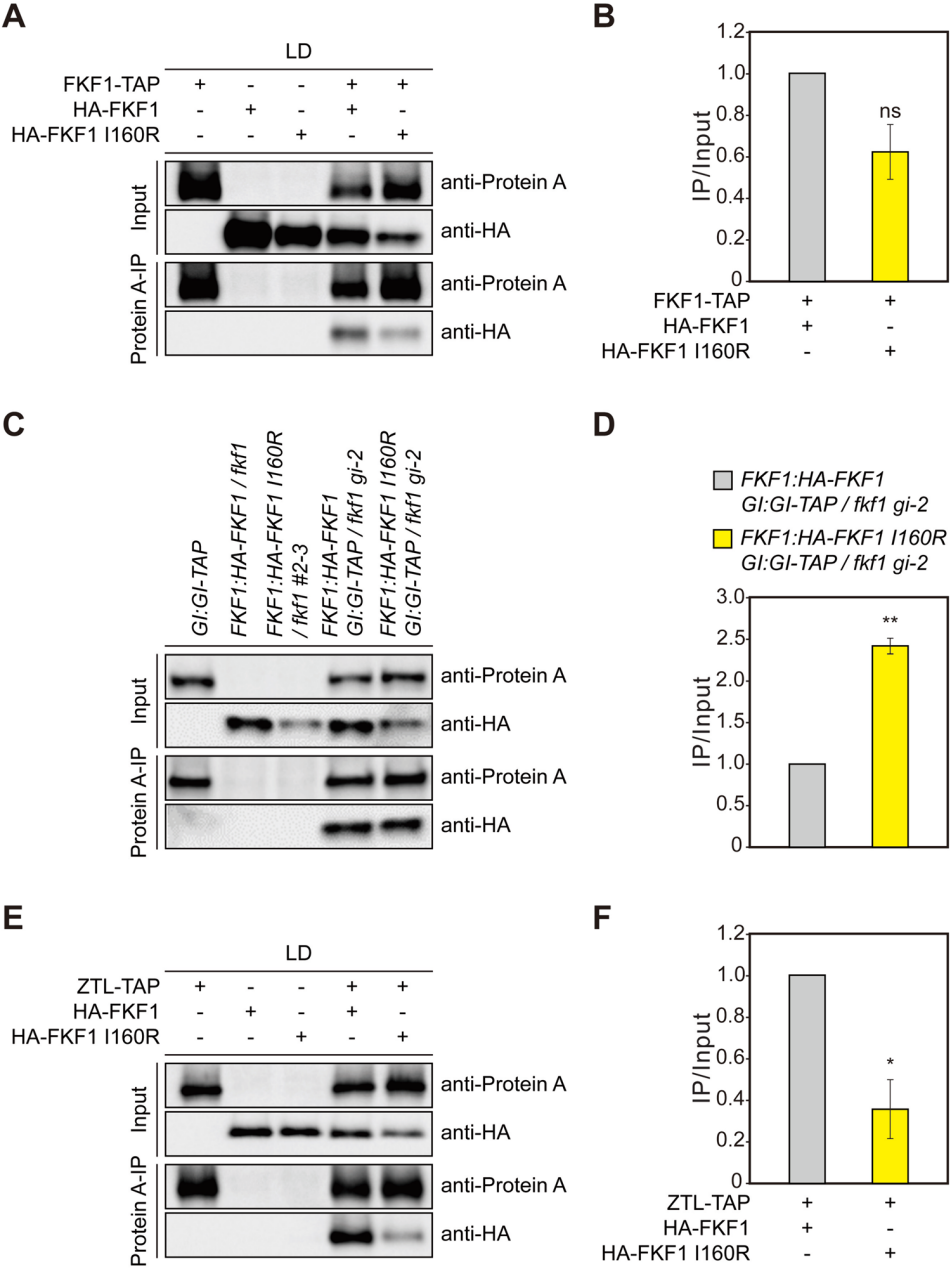
The I160R mutation influences FKF1 dimerization *in planta*. Co-immunoprecipitation (co-IP) assays using proteins expressed in leaves of *Nicotiana benthamiana* (**A**, **B**, **E**, **F**) and Arabidopsis transgenic lines (**C**, **D**). TAP-tagged proteins were immunoprecipitated with anti-Protein A antibody. Relative amounts of complex formation were quantified. **A**, **C**, **E** Representative images of the results of co-IP assays. **B**, **D**, **F** Bar graphs representing the amounts of co-precipitated HA-tagged proteins calculated as (HA-tagged protein_IP_/TAP-tagged protein_IP_)/(HA-tagged protein_Input_/TAP-tagged protein_Input_). **A**, **B** FKF1-TAP proteins were coexpressed with either HA-FKF1 or HA-FKF1 I160R protein in 3-week-old tobacco plants. **C**, **D** Arabidopsis plants were grown on agar medium under LDs and harvested at ZT12 on day 10. **E**, **F** ZTL-TAP proteins were transiently coexpressed with either HA-FKF1 or HA-FKF1 I160R in 3-week-old tobacco plants. The data underwent statistical analysis using a two-tailed Student's *t* test. *p < 0.05 and **p ≤ 0.01. *ns* not significant. Means ± SEM were calculated from three biological replicates

To substantiate the reduced FKF1 homodimerization observed in vitro, we transiently overexpressed TAP-tagged FKF1 (FKF1-TAP) with either HA-FKF1 or the HA-FKF1 I160R variant in *Nicotiana benthamiana* leaves under LDs. Co-immunoprecipitation (co-IP) revealed a decrease in the binding affinity of FKF1-TAP to the HA-FKF1 I160R variant compared to HA-FKF1 (Fig. 3A, B). Next, we asked whether the I160R mutation influences the complex formation of FKF1 with GI. We generated *FKF1:HA-FKF1 I160R GI:GI-TAP/fkf1* transgenic plants and subjected them to co-IP assays. Under LDs, the interaction between the FKF1 I160R variant and GI significantly increased compared to that between FKF1 and GI (Fig. 3C, D). As the LOV domain of FKF1 exists as a monomer when bound to GI (Kwon et al. 2022), these results suggest that the I160R mutation might facilitate the conversion of FKF1 homodimers to monomers, promoting subsequent GI binding. As FKF1 interacts with GI in a blue-light-dependent manner, we evaluated the impact of light quality on this interaction. Although some variations were observed, a similar trend in complex formation between FKF1–GI and FKF1 I160R–GI was observed under white-, red-, and blue-light conditions (Supplementary Fig. S3). These results suggest that the I160R mutation has minimal effects on the blue-light-mediated interaction.

In contrast to GI, ZTL, a homolog of FKF1, inhibits the function of FKF1 in regulating flowering by directly binding to this protein (Takase et al. 2011; Hwang et al. 2019). Thus, we examined whether the increased levels of the monomeric form of FKF1 affect its interaction with ZTL. In tobacco leaf cells, the amount of the FKF1 I160R variant co-immunoprecipitated with ZTL-TAP was significantly reduced compared to FKF1 (Fig. 3E, F), implying that ZTL may interact with the FKF1 homodimer. Collectively, these findings suggest that the conversion of FKF1 homodimers to monomers could play a pivotal role in promoting the levels of *FT* mRNA by modulating complex formation with *FT* regulators such as GI and ZTL.

### The FKF1 I160R variant enhances CO stability through increased binding to COP1

**Fig. 4:**
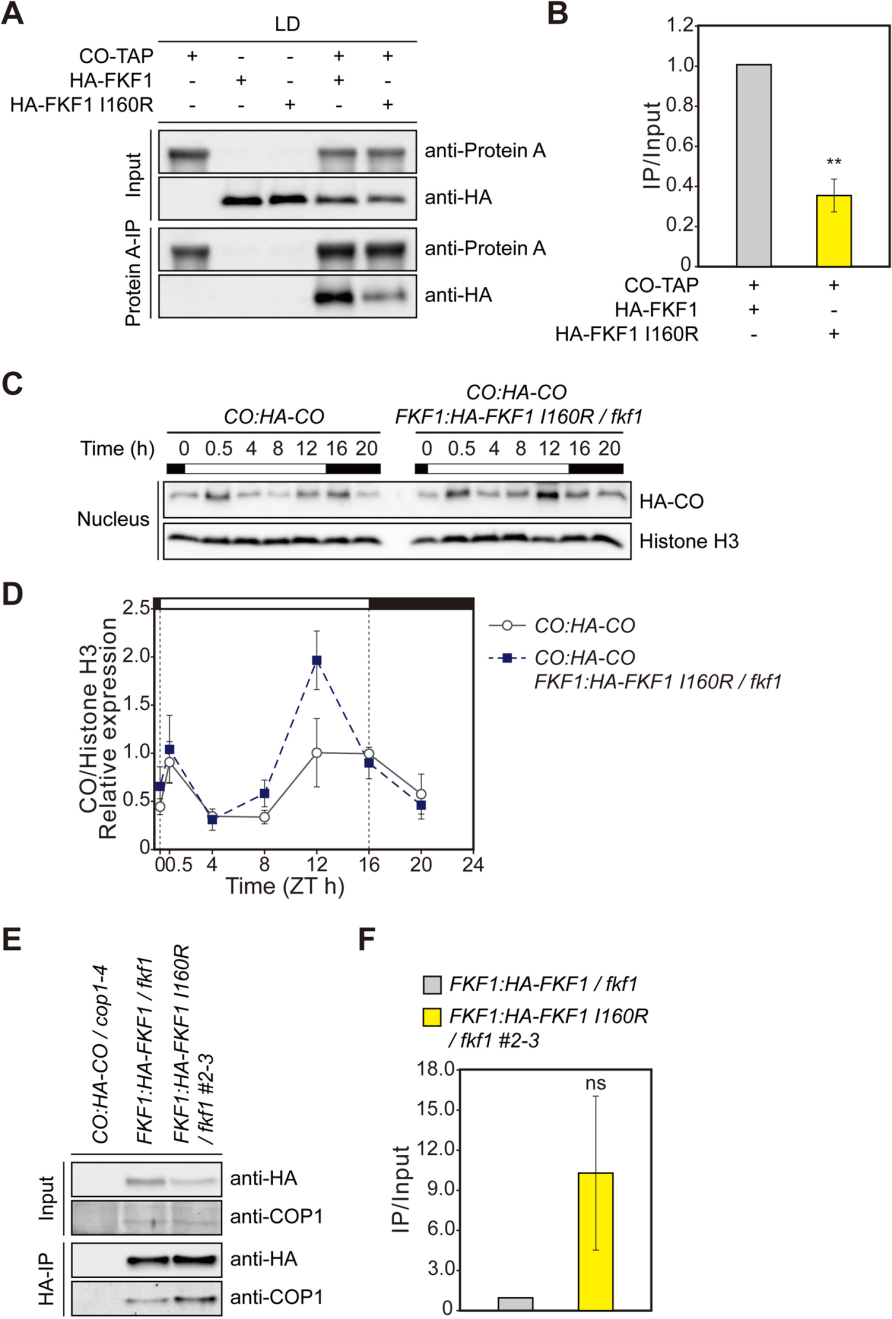
The FKF1 I160R variant shows enhanced binding affinity to COP1. **A**, **C**, **E** Representative images of the results of co-IP assays (**A**, **C**) and protein expression analysis (**E**). **B**, **D**, **F** Relative amounts of complex formation (**B**, **F**) and protein abundance (**D**) were quantified. **A**, **B** CO-TAP proteins were coexpressed with either HA-FKF1 or HA-FKF1 I160R in leaf cells of 3-week-old tobacco plants. The data underwent statistical analysis using a two-tailed Student's *t* test. **p ≤ 0.01. **C**, **D** *CO:HA-CO* and *CO:HA-CO FKF1:FKF1 I160R/fkf1* plants were grown under LDs and harvested at the indicated time points on day 10. Relative protein abundance was normalized to Histone H3 levels. Histone H3 protein was detected by immunoblotting with anti-Histone H3 antibody. **E**, **F** Seedlings were grown under LDs and sampled at ZT12 on day 10. **E** HA-tagged proteins were immunoprecipitated with anti-HA antibody. Endogenous COP1 proteins were detected by immunoblotting with anti-COP1 antibody. **F** Bar graphs representing the amounts of co-immunoprecipitated COP1 protein calculated as (COP1_IP_/HA-tagged protein_IP_)/(COP1_Input_/HA-tagged protein_Input_). The data underwent statistical analysis using a two-tailed Student's *t* test. *ns* not significant. The means ± SEM of normalized values calculated from three biological replicates are shown

CO serves as the primary *FT* activator, and FKF1 stabilizes CO in the afternoon through a direct interaction via its LOV domain (Samach et al. 2000; Song et al. 2012). Gene expression analysis showed that the FKF1 I160R variant selectively enhances *FT* expression over *CO* (Fig. 2E–H). This finding prompted us to hypothesize that the increased levels of FKF1 monomers resulting from the I160R mutation might increase the interaction of FKF1 with CO and/or enhance CO stability. To explore these possibilities, we carried out co-IP assays using proteins transiently overexpressed in tobacco leaves. Unexpectedly, the binding of CO to the FKF1 I160R variant was significantly reduced compared to its binding to FKF1 (Fig. 4A, B). We then analyzed CO protein levels using *CO:HA-CO* and *CO:HA-CO FKF1:FKF1-I160R/fkf1* lines. Under LDs, the daily expression profile of CO in the *CO:HA-CO FKF1:HA-FKF1 I160R/fkf1* line showed enhanced protein abundance in the afternoon, particularly at ZT12 (Fig. 4C, D). These results suggest that the FKF1 I160R variant indirectly upregulates CO stability rather than complex formation. COP1 is the main E3 ubiquitin ligase that is responsible for CO degradation throughout the day (Jang et al. 2008; Liu et al. 2008; Sarid-Krebs et al. 2015). Under LDs, FKF1 suppresses COP1 homodimerization by directly binding to it in the presence of light, thereby potentially diminishing COP1 activity and increasing the stabilization of CO in the afternoon (Lee et al. 2017). Therefore, we reasoned that the I160R mutation in FKF1 may increase its binding to COP1. We performed co-IP analysis using *FKF1:HA-FKF1/fkf1* and *FKF1:HA-FKF1 I160R/fkf1 #2–3* plants grown under LDs. The level of COP1 that co-precipitated with HA-FKF1 I160R notably increased compared to FKF1 in the late afternoon (Fig. 4E, F). These findings suggest that the FKF1 monomer preferentially interacts with COP1, leading to increased CO abundance.

## Discussion

**Fig. 5:**
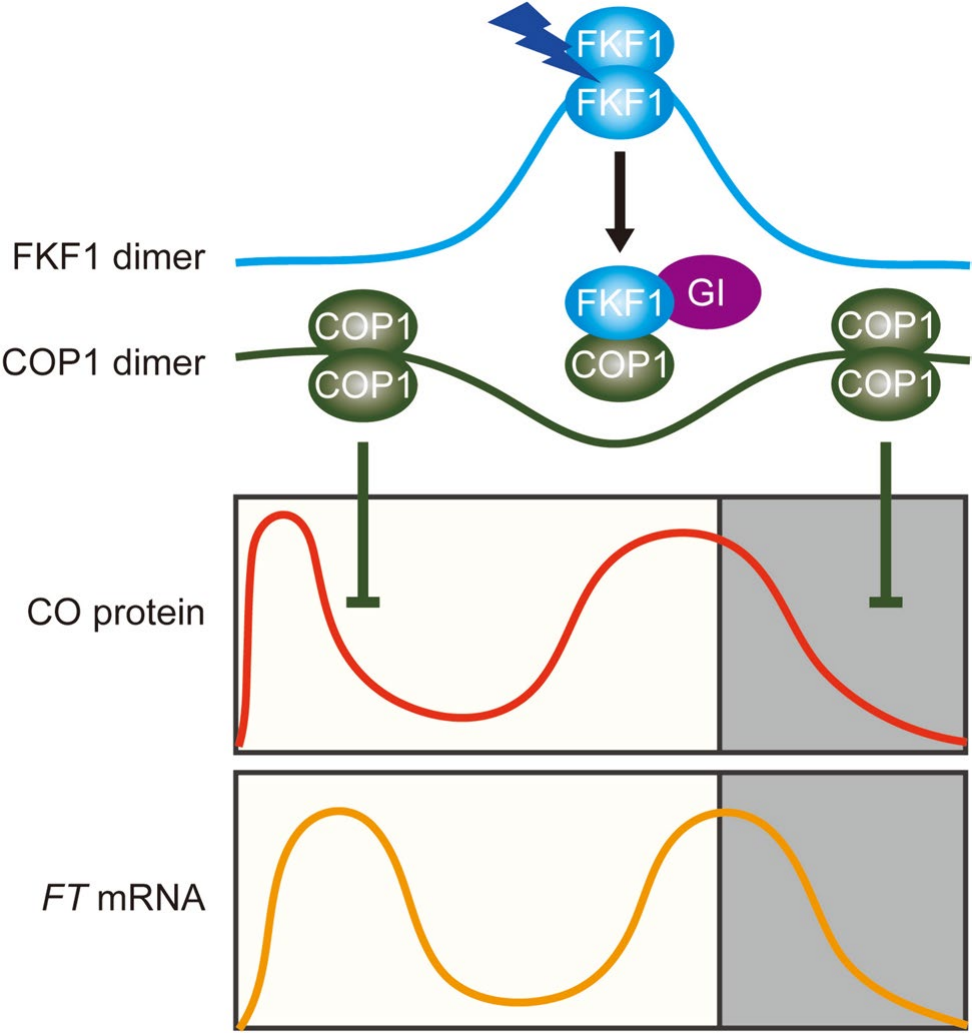
A hypothetical model describing the role of FKF1 dimerization in fine-tuning *FT* expression under long-day conditions. Under a long photoperiod, the abundance of CO protein exhibits dual peaks in the morning and late afternoon. In the morning, the COP1 dimer binds to CO and initiates its degradation via E3 ubiquitin ligase activity, resulting in reduced CO abundance. In the afternoon, the LOV-domain-containing blue-light photoreceptor FKF1 is expressed and naturally forms homodimers. The FKF1 homodimer interacts with and stabilizes CO, promoting its accumulation. Concurrently, the blue-light-activated FKF1 homodimer binds to GI through its LOV domain in the late afternoon. Upon GI binding, the FKF1 homodimer is converted to the monomeric form, causing a conformational change in its Kelch domain and adopting an open structure. Subsequently, the GI-bound FKF1 monomeric form interacts with COP1 through its Kelch domain, disrupting the COP1 homodimer. As a result, the COP1 monomer is unable to form a complex with SPA1 for CO degradation, leading to the significant accumulation of CO protein in the late afternoon. Consequently, the increased activity of CO induces peak levels of *FT* expression around dusk, thereby promoting flowering. During the night, due to impaired FKF1–GI complex formation, COP1 dimers accumulate and actively degrade CO

Many plants constantly monitor changes in daylength and thus coordinate the timing of the transition to the reproductive phase with favorable seasons, thereby maximizing seed production (Andrés and Coupland 2012; Song et al. 2015). In Arabidopsis, the blue-light photoreceptor FKF1 acts as the main photoperiod sensor involved in the photoperiod-dependent accumulation of *FT* transcript (Sawa et al. 2007; Song et al. 2012, 2015). In this study, using the FKF1 I160R variant, which shows attenuated homodimerization, we demonstrated that the regulation of FKF1 dimerization is important for optimizing the levels of *FT* induction in the afternoon under LDs (Fig. 5).

The interaction of FKF1 with GI is important for the ubiquitin ligase activity and stability of FKF1 (Sawa et al. 2007; Hwang et al. 2019). Under LDs, GI binding facilitates the function of FKF1 in the degradation of CDF1, allowing *CO* to be expressed in the afternoon (Sawa et al. 2007). However, the involvement of FKF1–GI complex formation in the induction of *FT* transcription remains poorly understood. In the current study, reducing the formation of FKF1 homodimers led to increased levels of *FT* mRNA but not *CO* mRNA (Figs. 2G, H and 3A, B), indicating that the conversion of FKF1 homodimers to monomers is crucial for the selective role of FKF1 in inducing *FT* expression. During the daytime under LDs, the FKF1 I160R variant demonstrated enhanced interactions with GI and COP1, resulting in elevated CO protein abundance (Figs. 3C, D and 4C–F). These findings may, at least in part, explain the heightened levels of *FT* mRNA observed in *FKF1:HA-FKF1 I160R/fkf1* plants around dusk under LDs (Fig. 2E–H). Notably, FKF1 protein accumulation appears to rely on the function of GI under LDs (Fornara et al. 2009; Hwang et al. 2019). Further investigation is needed to identify the factors contributing to the increased susceptibility of the monomeric form of FKF1 to degradation, despite its enhanced binding to GI (Figs. 2C, D and 4C, D).

The perception of blue light through the LOV domain is essential for the function of FKF1 in accelerating flowering time through interactions with not only GI but also CO (Imaizumi et al. 2003; Sawa et al. 2007; Song et al. 2012). While FKF1 interacts with GI in a blue-light-dependent manner using its LOV domain, the presence of blue light may not affect the conversion between the FKF1 homodimer and monomer (Sawa et al. 2007; Kwon et al. 2022). Conversely, GI binding appears to disrupt LOV domain homodimerization after photoactivation (Kwon et al. 2022). Our co-IP analysis revealed that even with the I160R mutation, FKF1 maintained its ability to bind to GI in a blue-light-dependent manner (Supplementary Fig. S3), supporting the previous observation (Kwon et al. 2022). Under LDs, FKF1 also binds to CO through the LOV domain, and this binding is enhanced by blue light (Song et al. 2012). However, in contrast to GI, the binding of FKF1 to CO was significantly reduced by the I160R mutation (Fig. 4A, B), indicating that FKF1 may interact with CO as a homodimer or the I160 site may lie within the FKF1-CO interface. Considering the promotion of flowering via elevated *FT* induction in *FKF1:HA-FKF1 I160R/fkf1* plants (Fig. 2G, H), the conversion of FKF1 to the monomeric form appears to offer several advantages in its function, which counteract the adverse effects of this conversion on protein stability and CO binding. The interaction between FKF1 I160R and ZTL significantly decreased compared to that between FKF1 and ZTL (Fig. 3E, F). Since ZTL inhibits FKF1 activity and competes with it for GI binding (Takase et al. 2011; Hwang et al. 2019), the reduced complex formation may contribute to the increased interaction of the FKF1 monomers with GI (Fig. 3C, D).

The interaction between ZTL and GI through the LOV domain results in diminished affinity towards its proteolytic targets, PRR5 and TOC1 (Kim et al. 2007). The formation of the ZTL–GI complex induces a conformational change in the Kelch domain, leading to a closed configuration (Kwon et al. 2022). This change might contribute to the increased abundance of PRR5 and TOC1 during the night (Kim et al. 2007). By contrast, when FKF1 interacts with GI in a blue-light-specific manner, the Kelch domain appears to adopt an open configuration, allowing it to recognize CDF1, a substrate for the E3 ubiquitin ligase activity of FKF1 (Imaizumi et al. 2005; Sawa et al. 2007; Fornara et al. 2009; Kwon et al. 2022). Under LDs, FKF1 binds to the COP1 monomer under blue light through the Kelch domain (Lee et al. 2017). However, unlike CDF1, COP1 protein abundance is unaffected by the mutation or overexpression of *FKF1* (Lee et al. 2017). Instead, FKF1 binding inhibits COP1 homodimerization, which mediates the degradation of CO by forming a tetramer complex with SPA1 (Laubinger et al. 2006; Jang et al. 2008; Zhu et al. 2008; Lee et al. 2017). Consistent with this finding, our data show that the enhanced interaction of FKF1 with COP1 due to the I160R mutation led to increased CO protein abundance (Fig. 4C–F). Considering the enhanced binding affinity of FKF1 to GI due to the I160R mutation (Fig. 3C, D), these findings support the notion that GI binding may facilitate the dissociation of FKF1 homodimers and the complex formation of FKF1 monomers with COP1, allowing CO to accumulate in the afternoon under LDs by decreasing COP1–SPA1 complex formation (Fig. 5). Furthermore, given that COP1 destabilizes GI in conjunction with ELF3 (Yu et al. 2008), the heightened formation of the FKF1 and COP1 complex due to the I160R mutation could potentially enhance GI accumulation. This, in turn, might enhance the abundance of *FT* mRNA via the GI-mediated microRNA172 pathway, independently of *CO* transcription (Jung et al. 2007).

In addition to regulating CO stability, FKF1 may directly induce *FT* expression through GI binding. Both FKF1 and GI associate with the *FT* promoter, and FKF1 relieves *FT* repression by CDF1 (Sawa et al. 2007; Song et al. 2012), indicating that FKF1 likely forms a complex with GI at the *FT* locus. Moreover, GI interacts with *FT* repressors, such as SHORT VEGETATIVE PHASE (SVP) and TEMPRANILLOs (TEMs) (Sawa et al. 2007). As FKF1 and GI bind to the *FT* promoter regions near its SVP-binding sites (Lee et al. 2007; Sawa et al. 2007; Song et al. 2012), perhaps FKF1 is involved in reducing *FT* repression by SVP and TEMs by forming a complex with GI. Together, our findings suggest that the regulation of FKF1 dimerization provides a new layer that determines the optimal levels of *FT* expression during the afternoon. This regulatory mechanism might enable plants to finely adjust their flowering time in response to dynamic environmental fluctuations.

### Supplementary Information

Below is the link to the electronic supplementary material.

Supplementary file1 (DOCX 4576 KB)

## Data Availability

The data supporting the findings of this study are provided in the main text and supplementary materials.
