# Supplementary material for: Disrupting FKF1 homodimerization increases FT transcript levels in the evening by enhancing CO stabilization
Source: Plant Cell Rep. 2024 Apr 18;43(5):121. doi: 10.1007/s00299-024-03207-w (PMC11026275; doi:10.1007/s00299-024-03207-w)
Supplement: Supplementary file 1 — Supplementary file1 (DOCX 4576 KB) [file 299_2024_3207_MOESM1_ESM.docx]

***Supplementary Information***

**Title:** **Disrupting FKF1 homodimerization increases *FT* transcript levels in the evening by enhancing CO stabilization**

**Authors:** Sung Won Cho^1,2^, Jameela Lokhandwala^3^, Jun Sang Park^4^, Hye Won Kang^4^, Mingi Choi^4^, Hong-Quan Yang^5^, Takato Imaizumi^6^, Brian D Zoltowski^3^, and Young Hun Song^2,4,7,*^

**Affiliations:**

^1^Department of Biology, Ajou University, Suwon, Korea

^2^Institute of Agricultural Life Sciences, Seoul National University, Seoul, Korea

^3^Department of Chemistry, Southern Methodist University, Dallas, TX, USA

^4^Department of Agricultural Biotechnology, Seoul National University, Seoul, Korea

^5^Shanghai Key Laboratory of Plant Molecular Sciences, College of Life Sciences, Shanghai Normal University, Shanghai, China

^6^Department of Biology, University of Washington, Seattle, WA, USA

^7^Plant Genomics and Breeding Institute, Seoul National University, Seoul, Korea

^*^Correspondence to: younghsong@snu.ac.kr, 1 Gwanak-ro, Gwanak-gu, Seoul 08826, Korea, +82-2-880-4649

# Supplementary Figures


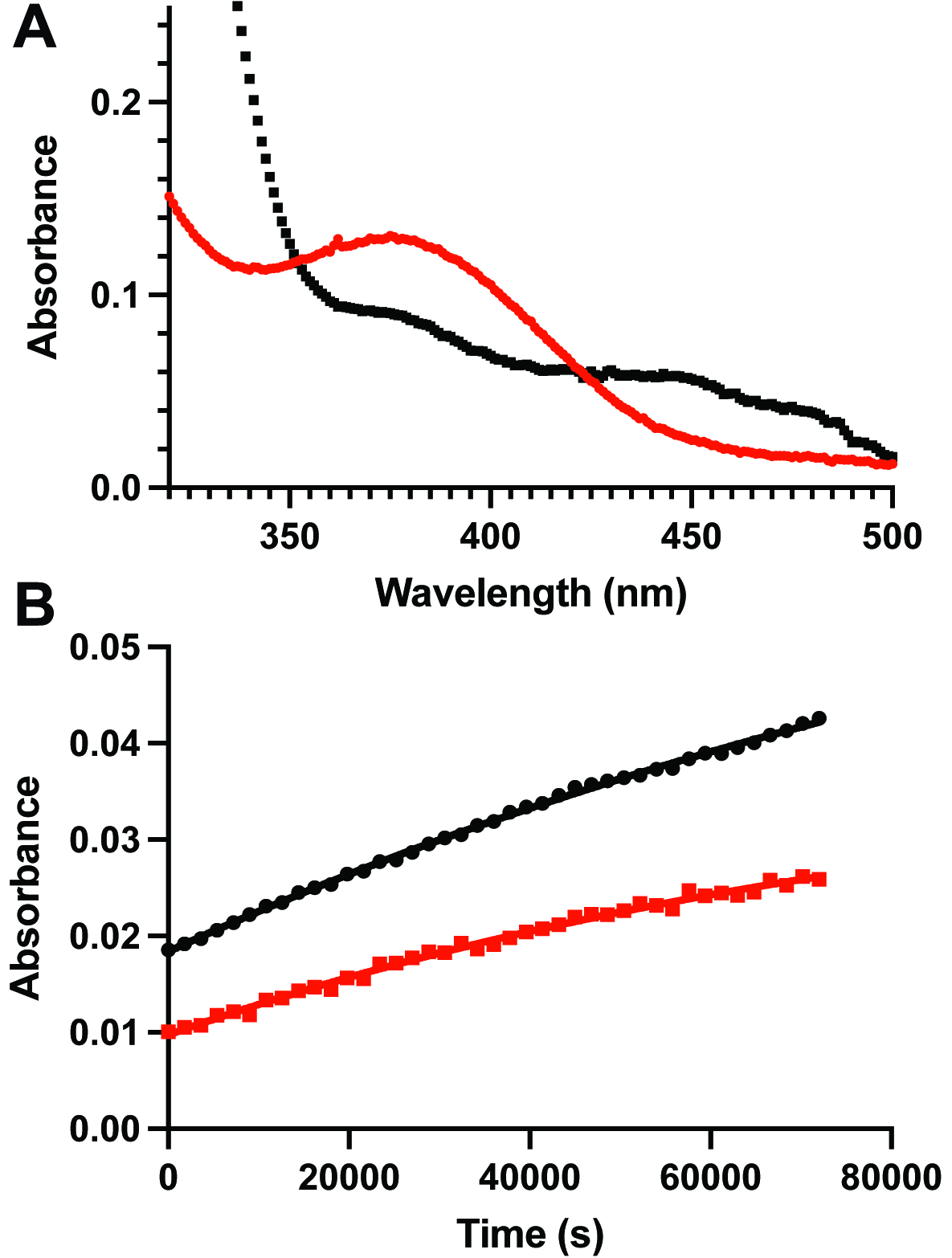


**Fig. S1** Photochemistry and Kinetics of the FKF1 I160R variant. **A** The FKF1 I160R variant expressed and purified in the light demonstrates a spectra consistent with the light state of LOV proteins (red), with a single peak at 380 nm indicative of the formation of a Flavin-C4a adduct. Incubation in the dark for 24 hr, in the presence of 300 mM imidazole as a base catalyst, results in the return of a dark state peak at 450 nm. Complete conversion to the dark state is complicated by the extremely slow dark-state reversion kinetics in FKF1 proteins (Pudasaini and Zoltowski 2013). **B** Absorbance at 450 nm (black) and 478 nm (red) collected every 1800 sec. The solid line represents a single exponential fit to the time course data. Kinetic analysis of the FKF1 I160R variant in the presence of 300 mM imidazole demonstrates a slow dark state recovery (k_T_=8x10^-6^ s^-1^ +/- 1.3x10^-6^), consistent with WT proteins (FKF1@300 mM imidazole: k_T_=6.5x10^-6^ s^-1^ +/- 3x10^-7^). The error in rate constants is based on the 95% Confidence Interval from the single exponential fit at 450 nm for the FKF1 I160R variant. For FKF1, the error represents the standard deviation from data recorded in triplicate.


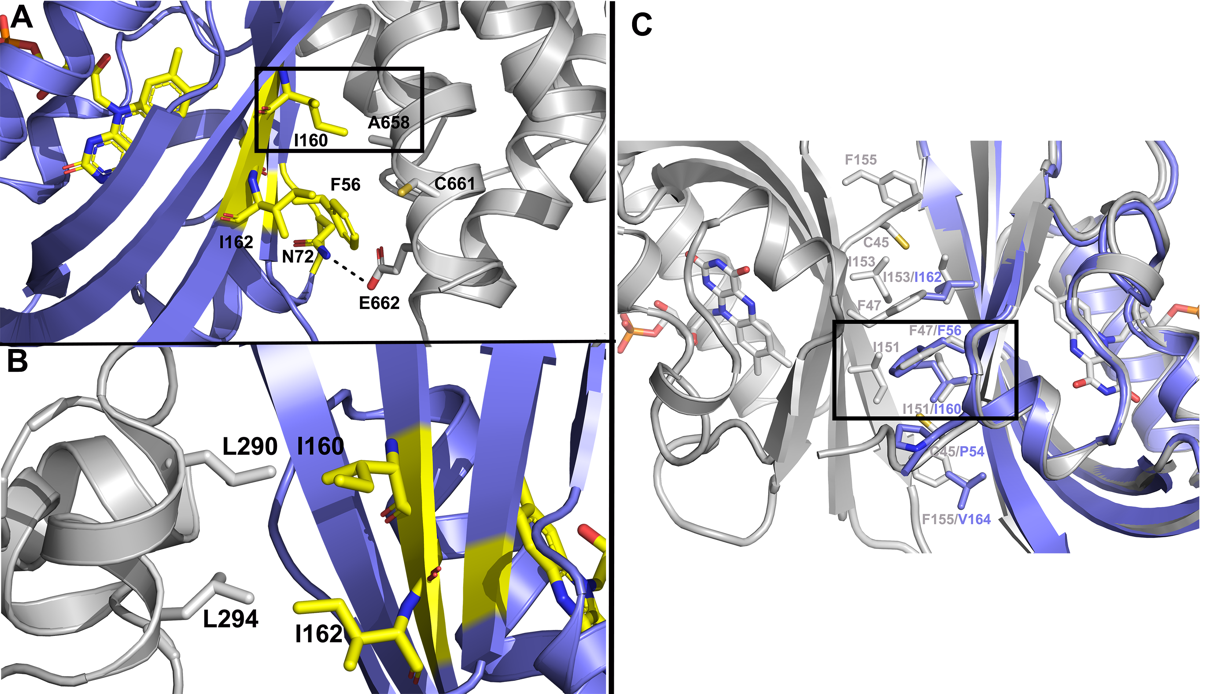


**Fig. S2** Structural Predictions for FKF1:GI and FKF1:ZTL Interactions. Structures above are based on Alphafold (AF2) (Jumper et al. 2021) predictions for the structure of FKF1. The LOV domain from the AF2-FKF1 structure (blue) was templated onto the GI:LKP2 structure (Grey) (PDBID: 7WA4) (Panels A and B) and ZTL (PDBID: 5SVG) (Panel C) using the SSM Superpose function in Coot (Emsley and Cowtan 2004). **A** Based on the proposed GI:LKP2 dimer (Kwon et al. 2022), I160 lies (black box) at a solvent-exposed site within the predicted GI:FKF1 heterodimer. The solvent-exposed site, in conjunction with the small side chain of A658 nearby, permits the I160R substitution without disruption of the GI:FKF1 interface. Other equivalent residues important to the ZTL dimer (I162 and F56) lie at the solvent-exposed site. Further, an H-bonding interaction between N72 (FKF1) and E662 (GI), which was identified as important for GI:LKP2 is conserved (Kwon et al. 2022). **B** The I160 side chain primarily makes contacts with a symmetry-related GI molecule within the GI:LKP2 structure. The alternative interface has a significantly lower buried surface area calculated with PISA (Krissinel and Henrick, 2007) (516.8 Å^2^ vs. 856.9 Å^2^) making it unlikely to represent a biologically relevant interface although this weaker interface may explain previous yeast-2-hybrid studies suggesting interactions with a 1-391 fragment of GI (Sawa et al., 2007). Interestingly, I160 and I162 of FKF1 contact L290 and L294 of GI in a manner that mimics the native ZTL dimer interface (Panel **C**), where I160R variants abolish dimerization. **C** The ZTL homodimer (grey) is defined by an extensive hydrophobic surface mediated by I151 and I153 (I160 and 162 in FKF1). Additional π-π and sulfur-π interactions stabilize the interface and are conserved in both ZTL and FKF1 (blue). The introduction of I151R (ZTL) and I160R (FKF1) variants has been shown to disrupt the dimerization of homodimers (Pudasaini et al. 2017). Due to the conservation of the residues within the dimer interface, I160R variants would disrupt ZTL:FKF1 heterodimers.

#
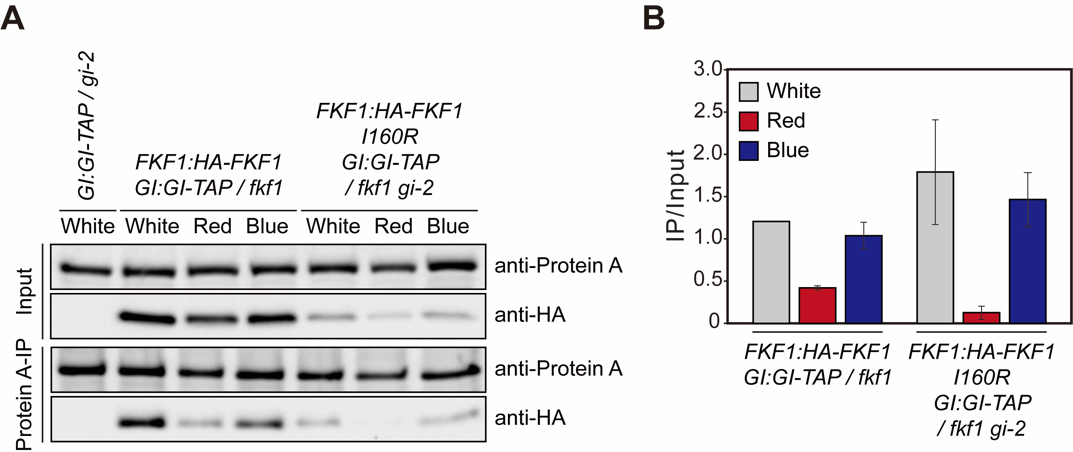


# Fig. S3 The effects of light quality on complex formation between the FKF1 I160R variant and GI. Seedlings grown on agar medium under LDs were kept under LDs (white) or transferred to monochromatic red (660 nm) or blue (450 nm) light on day 8 and harvested at ZT12 on day 10. GI-TAP proteins were immunoprecipitated with anti-Protein A antibody, and the presence of HA-FKF1 was detected by immunoblotting with anti-HA antibody. A The results of two biological replicates of co-immunoprecipitation (co-IP) assays. B Bar graphs representing the amounts of co-immunoprecipitated HA-tagged proteins calculated as (HA-tagged protein_IP_/GI-TAP_IP_)/(HA-tagged protein_Input_/GI-TAP_Input_). Similar results were obtained in two biological replicates.
